# Improving Adversarial Robustness via Attention and Adversarial Logit Pairing

**DOI:** 10.3389/frai.2021.752831

**Published:** 2022-01-27

**Authors:** Xingjian Li, Dou Goodman, Ji Liu, Tao Wei, Dejing Dou

**Affiliations:** ^1^ Big Data Lab, Baidu Research, Beijing, China; ^2^ X-Lab, Baidu Inc., Beijing, China

**Keywords:** adversarial training, attention, adversarial robustness, adversarial example, deep learning, deep neural network

## Abstract

Though deep neural networks have achieved the state of the art performance in visual classification, recent studies have shown that they are all vulnerable to the attack of adversarial examples. In this paper, we develop improved techniques for defending against adversarial examples. First, we propose an enhanced defense technique denoted **Attention and Adversarial Logit Pairing (AT + ALP)**, which encourages both attention map and logit for the pairs of examples to be similar. When being applied to clean examples and their adversarial counterparts, **AT + ALP** improves accuracy on adversarial examples over adversarial training. We show that **AT + ALP** can effectively increase the average activations of adversarial examples in the key area and demonstrate that it focuses on discriminate features to improve the robustness of the model. Finally, we conduct extensive experiments using a wide range of datasets and the experiment results show that our **AT + ALP** achieves **the state of the art** defense performance. For example, on **17 Flower Category Database**, under strong 200-iteration Projected Gradient Descent (PGD) gray-box and black-box attacks where prior art has 34 and 39% accuracy, our method achieves **50** and **51%**. Compared with previous work, our work is evaluated under highly challenging PGD attack: the maximum perturbation *ϵ* ∈ {0.25, 0.5} i.e. *L*_*∞*_ ∈ {0.25, 0.5} with 10–200 attack iterations. To the best of our knowledge, such a strong attack has not been previously explored on a wide range of datasets.

## 1 Introduction

In recent years, deep neural networks have been extensively deployed for computer vision tasks, particularly for visual classification problems, where new algorithms have been reported to achieve even better performance than human beings Krizhevsky et al. (2012), He et al. (2015), Li et al. (2019a). The success of deep neural networks has led to an explosion in demand. However, recent studies have shown that they are all vulnerable to the attack of adversarial examples Szegedy et al. (2013); Carlini and Wagner (2016); Moosavi-Dezfooli et al. (2016); Bose and Aarabi (2018). Small and often imperceptible perturbations to the input images are sufficient to fool the most powerful deep neural networks.

**FIGURE 1:**
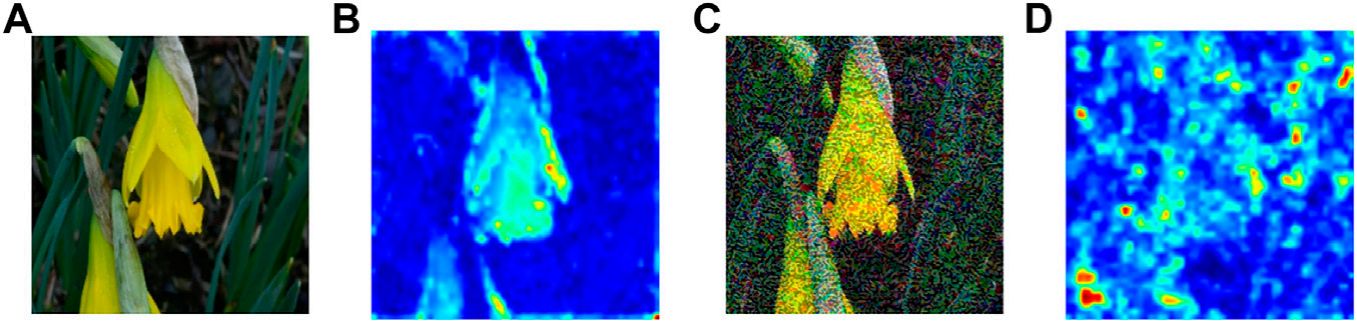
**(A)** is original image and **(B)** is corresponding spatial attention map of ResNet-50 He et al. (2015) pretrained on ImageNet Russakovsky et al. (2015) which shows where the network focuses in order to classify the given image. **(C)** is adversarial image of **(A)**, **(D)** is corresponding spatial attention map.

In Figure 1, we visualize the spatial attention map of a flower and its corresponding adversarial image on ResNet-50 He et al. (2015) pretrained on ImageNet Russakovsky et al. (2015). The figure suggests that adversarial perturbations, while small in the pixel space, lead to very substantial “noise” in the attention map of the network. Whereas the features for the clean image appear to focus primarily on semantically informative content in the image, the attention map for the adversarial image are activated across semantically irrelevant regions as well. The state of the art adversarial training methods only encourage hard labels Madry et al. (2017); Tramèr et al. (2017) or logit Kannan et al. (2018) for pairs of clean examples and adversarial counterparts to be similar. In our opinion, it is not enough to align the difference between the clean examples and adversarial counterparts only at the end part of the whole network, i.e., hard labels or logit, and we need to align the attention maps for important parts of the whole network. Motivated by this observation, we explore **Attention and Adversarial Logit Pairing(AT + ALP)**, a method that encourages both attention map and logit for pairs of examples to be similar. When being applied to clean examples and their adversarial counterparts, **AT + ALP** improves accuracy on adversarial examples over adversarial training.

The contributions of this paper are summarized as follows:

• We introduce enhanced adversarial training using a technique we call **Attention and Adversarial Logit Pairing(AT + ALP)**, which encourages both attention map and logit for pairs of examples to be similar. When being applied to clean examples and their adversarial counterparts, **AT + ALP** improves accuracy on adversarial examples over adversarial training.
• We show that our **AT + ALP** can effectively increase the average activations of adversarial examples in the key area and demonstrate that it focuses on more discriminate features to improve the robustness of the model.
• We show that our **AT + ALP** achieves **the state of the art** defense on a wide range of datasets against strong **PGD** gray-box and black-box attacks. Compared with previous work, our work is evaluated under highly challenging PGD attack: the maximum perturbation *ϵ* ∈ {0.25, 0.5}, i.e., *L*_*∞*_ ∈ {0.25, 0.5} with 10–200 attack iterations. To the best of our knowledge, such a strong attack has not been previously explored on a wide range of datasets.

The rest of the paper is organized as follows: in Section 2, we present the related works; in Section 3, we introduce definitions and threat models; in Section 4 we propose our **Attention and Adversarial Logit Pairing(AT + ALP)** method; in Section 5, we show extensive experimental results; and Section 6 concludes.

## 2 Related Work

Athalye et al. (2018) evaluate the robustness of nine papers Buckman et al. (2018); Ma et al. (2018); Guo et al. (2017); Dhillon et al. (2018); Xie et al. (2017); Song et al. (2017); Samangouei et al. (2018); Madry et al. (2017); Na et al. (2017) accepted by ICLR 2018 as non-certified white-box-secure defenses to adversarial examples. They find that seven of the nine defenses use obfuscated gradients, a kind of gradient masking, as a phenomenon that leads to a false sense of security in defenses against adversarial examples. Obfuscated gradients provide a limited increase in robustness and can be broken by improved attack techniques they develop. The only defense they observe that significantly increases robustness to adversarial examples within the threat model proposed is **adversarial training** Madry et al. (2017).

Adversarial training Goodfellow et al. (2015); Madry et al. (2017); Kannan et al. (2018); Tramèr et al. (2017); Pang et al. (2019) defends against adversarial perturbations by training networks on adversarial images that are generated on-the-fly during training. For adversarial training, the most relevant work to our study is Kannan et al. (2018), which introduce a technique they call **Adversarial Logit Pairing (ALP)**. This method encourages logits for pairs of examples to be similar. Our **AT + ALP** encourages both attention map and logit for pairs of examples to be similar. When being applied to clean examples and their adversarial counterparts, **AT + ALP** improves accuracy on adversarial examples over adversarial training. Araujo et al. (2019) adds random noise at training and inference time, Xie et al. (2018) adds denoising blocks to the model to increase adversarial robustness, while neither of the above approaches focuses on the attention map.

In terms of methodologies, our work is also related to deep transfer learning and knowledge distillation problems, and the most relevant work to our study is Zagoruyko and Komodakis (2016); Li et al. (2019b), which constrain the *L*_2_-norm of the difference between their behaviors (i.e., the feature maps of outer layer outputs in the source/target networks). Our **AT + ALP** constrains attention map and logit for pairs of clean examples and their adversarial counterparts to be similar.

## 3 Definitions and Threat Models

In this paper, we always assume the attacker is capable of forming attacks that consist of perturbations of limited *L*_*∞*_-norm. This is a simplified task chosen because it is more amenable to benchmark evaluations. We consider two different threat models characterizing amounts of information the adversary can have:

• **Gray-box Attack** We focus on defense against gray-box attacks in this paper. In a gray-back attack, the attacker knows both the original network and the defense algorithm. Only the parameters of the defense model are hidden from the attacker. This is also a standard setting assumed in many security systems and applications Pfleeger and Pfleeger (2004).
• **Black-box Attack** The attacker has no information about the model’s architecture or parameters, and no ability to send queries to the model to gather more information.

## 4 Methods

### 4.1 Architecture

**FIGURE 2:**
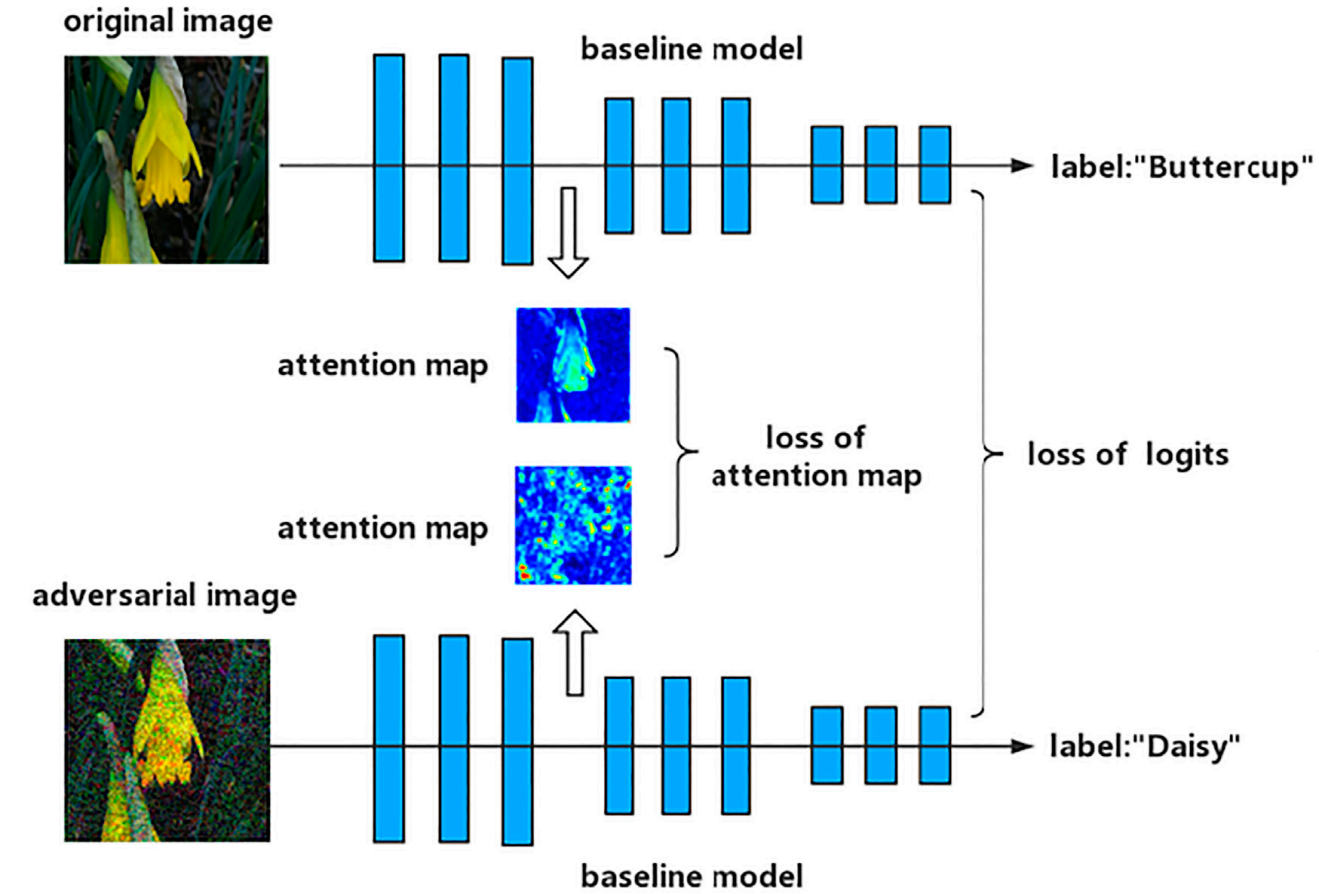
Schematic representation of **Attention and Adversarial Logit Pairing (AT + ALP)**: a baseline model is trained so as, not only to make similar logits, but to also have similar spatial attention maps to those of original image and adversarial image.

Figure 2 represents architecture of **Attention and Adversarial Logit Pairing (AT + ALP)**: a baseline model is adversarial trained so as, not only to make similar logits, but to also have similar spatial attention maps to those of original image and adversarial image.

### 4.2 Adversarial Training

We use adversarial training with **Projected Gradient Descent (PGD)** Madry et al. (2017) as the underlying basis for our methods:

$$\underset{\theta }{\mathrm{arg min}}E_{\left(x,y\right)\in {\hat{p}}_{\text{ data }}}\left(\underset{\delta \in S}{\max}L\left(\theta ,x+\delta ,y\right)\right)$$ (1)

where ${\hat{p}}_{\text{ data }}$ is the underlying training data distribution, *L* (*θ*, *x* + *δ*, *y*) is a loss function at data point *x* which has true class *y* for a model with parameters *θ*, and the maximization with respect to *δ* is approximated using PGD. In this paper, the loss is defined as:

$$L=L_{CE}+\alpha L_{ALP}+\beta L_{AT},$$ (2)

where *L*_*CE*_ is cross entropy, *α* and *β* are hyperparameters.

### 4.3 Adversarial Logit Pairing

We also use **Adversarial Logit Pairing (ALP)** to encourage the logits from clean examples and their adversarial counterparts to be similar to each other. For a model that takes inputs *x* and computes a vector of logit *z* = *f*(*x*), logit pairing adds a loss:

$$L_{ALP}=L_{a}\left(f\left(x\right),f\left(x+\delta \right)\right)$$ (3)

In this paper we use *L*_2_ loss for *L*_*a*_.

### 4.4 Attention Map

We use **Attention Map (AT)** to encourage the attention map from clean examples and their adversarial counterparts to be similar to each other. Let *I* denote the indices of all activation layer pairs, for which we want to pay attention. Then, we can define the following total loss:

$$L_{AT}=\underset{j\in I}{\sum }{\left\|\frac{Q_{ADV}^{j}}{{\left\|Q_{ADV}^{j}\right\|}_{2}}-\frac{Q_{O}^{j}}{{\left\|Q_{O}^{j}\right\|}_{2}}\right\|}_{p}$$ (4)

Let *O*, *ADV* denote clean examples and their adversarial counterparts, where $Q_{O}^{j}=\mathrm{vec}\left(F\left(A_{O}^{j}\right)\right)$ and $Q_{ADV}^{j}=\mathrm{vec}\left(F\left(A_{ADV}^{j}\right)\right)$ are respectively the *j*th pair of clean examples and their adversarial counterparts attention maps in vectorized form, and *p* refers to norm type (in the experiments we use *p* = 2).

### 4.5 Experiments: White-Box Settings

White-box attack is the most challenging task for evaluating a model’s adversarial robustness. In white-box settings, attackers are assumed to know all details about the model, including its architecture and parameters. We conduct white-box experiments following common practices Madry et al. (2017); Kannan et al. (2018). Specifically, we use ResNet-18 He et al. (2015) trained with CIFAR-10 Krizhevsky and Hinton (2009).

We use Fast Gradient Sign (FGS) Goodfellow et al. (2015), Projected Gradient Descent (PGD) Madry et al. (2017), AutoAttack Croce and Hein (2020) and RayS Chen and Gu (2020) to perform white-box attacks towards evaluated models. We consider untargeted attack, which is more challenging for defense than targeted attack. Adversarial perturbations are measured by *L*_*∞*_ norm (i.e., maximum perturbation for each pixel), with an allowed maximum value of *ϵ* = 8/255.

### 4.6 Image Database

The CIFAR-10 Krizhevsky and Hinton (2009) dataset contains 50,000 training samples and 10,000 test samples, uniformly distributed across 10 classes. Each sample is a 32 × 32 color image. Though with a low image resolution, CIFAR-10 is a popular benchmark to evaluate the adversarial robustness of a model.

### 4.7 Experimental Setup

For white-box settings, we use ResNet-18 He et al. (2015) as the model architecture. Models are first trained on CIFAR-10 with different adversarial training methods, including PAT Madry et al. (2017), ALP Kannan et al. (2018), TRADES Zhang et al. (2019) and our proposed Attention Map (**AT**). We train all models with 100 epochs following practices suggested by TRADES Zhang et al. (2019). For adversarial attacks, we adopt 1-step FSG attack Goodfellow et al. (2015), 7-iteration PGD attack Madry et al. (2017) and AutoAttack Croce and Hein (2020) with the common used perturbation magnitude of *ϵ* = 8/255 under *L*_*∞*_ norm. We also evaluate them with RayS Chen and Gu (2020), which is a gradient-free adversarial attack requiring only the target model’s hard-label output. We run each experiments three times and report the average top-1 accuracy. We also report the training time of each method for a more comprehensive comparison. Our experiments are run on Nvidia Tesla V100-SXM2 GPUs.

## 5 Results and Discussion

We present results of the white-box experiment in Table 1. We compare the proposed Attention adversarial training (**AT**) against relevant methods including PAT Madry et al. (2017), ALP Kannan et al. (2018) and TRADES Zhang et al. (2019). As seen in Table 1, all of these methods show certain degree of robustness, even under the advanced adversarial attacks such as AutoAttack. Specifically, our **AT** is superior to baseline methods PAT and ALP, with higher clean accuracy, robust accuracy under FSG, PGD and AutoAttack. TRADES Zhang et al. (2019) improves ALP by involving an inner maximization to generate a most *different* counterpart for the clean example. Therefore, TRADES achieves higher adversarial accuracy than other methods. However, the drawback lies in its efficiency, i.e. TRADES is slower than other adversarial training methods by about %46. This is because TRADES needs 10 adversarial steps per batch to achieve good performance, while seven steps are enough for ALP and AT. Moreover, the proposed **AT** achieves the highest clean accuracy among all these adversarial training methods.

**TABLE 1:** Defense against white-box attack on CIFAR-10. The adversarial perturbations were produced using Fast Gradient Sign (FGS) Goodfellow et al. (2015), Projected Gradient Descent (PGD) Madry et al. (2017), AutoAttack (AA) Croce and Hein (2020) and RayS Chen and Gu (2020). The perturbation magnitude is *ϵ* = 8/255 under *L*_*∞*_ norm.

Defense on CIFAR-10 database	Clean	FGS	PGD	AA	RayS	Time (hours)
No Defence	95.3	< 1	< 1	< 1	< 1	0.4
PAT Madry et al. (2017)	83.2	55.7	51.6	46.1	57.3	2.6
ALP Kannan, Kurakin, and Goodfellow (2018)	82.7	56.4	52.7	46.8	59.4	2.6
Our **AT**	83.5	56.9	53.0	48.4	59.2	2.6
TRADES Zhang et al. (2019)	82.1	58.1	54.6	49.0	58.9	3.7

RayS Chen and Gu (2020) performs adversarial attack from a different perspective. As RayS is gradient-free and independent of certain adversarial losses, it can be used to detect possible falsely robust models, especially those may overfit to specific types of gradient-based attacks and adversarial losses. As seen in Table 1, all advanced adversarial training methods including AL, AT and TRADES, show higher robustness under RayS attack. Our results are consistent with those reported in RayS Chen and Gu (2020) that, when evaluated on really robust models, the robust accuracy of RayS is usually higher than that of standard PGD.

### 5.1 Experiments: Gray and Black-Box Settings

To evaluate the effectiveness of our defense strategy, we performed a series of image-classification experiments on **17 Flower Category Database** Nilsback and Zisserman (2006), **Part of ImageNet Database** and **Dogs-vs.-Cats Database**. Following Athalye et al. (2018); Xie et al. (2018), we assume an adversary that uses the state of the art PGD adversarial attack method.

We consider untargeted attacks when evaluating under the gray and black-box settings; untargeted attacks are also used in our adversarial training. We evaluate top-1 classification accuracy on validation images that are adversarially perturbed by the attacker. In this paper, adversarial perturbation is considered under *L*_*∞*_ norm. The value of *ϵ* is relative to the pixel intensity scale of 256, we use *ϵ* = 64/256 = 0.25 and *ϵ* = 128/256 = 0.5. PGD attacker with 10–200 attack iterations and step size *α* = 1.0/256 = 0.0039. Our baselines are ResNet-101/152. There are four groups of convolutional structures in the baseline model, group-0 extracts of low-level features, group-1 and group-2 extract of mid-level features, group-3 extracts of high-level features Zagoruyko and Komodakis (2016), which are described as *conv*2_*x*, *conv*3_*x*, *conv*4_*x* and *conv*5_*x* in He et al. (2015).

### 5.2 Image Database

We performed a series of image-classification experiments on a wide range of datasets.

• **17 Flower Category Database** Nilsback and Zisserman (2006) contains images of flowers belonging to 17 different categories. The images were acquired by searching the web and taking pictures. There are 80 images for each category.
• **Part of ImageNet Database** contains images of four objects. These four objects are randomly selected from the ImageNet Database Russakovsky et al. (2015). In this experiment, they are tench, goldfish, white shark and dog. Each object contains 1,300 training images and 50 test images.
• **Dogs-vs.-Cats Database** ^1^ contains 8,000 images of dogs and cats in the train dataset and 2,000 in the test val dataset.

### 5.3 Experimental Setup

To perform image classification, we use ResNet-101/152 that were trained on the **17 Flower Category Database**, **Part of ImageNet Database** and **Dogs-vs.-Cats Database** training set. We consider two different attack settings: 1) a gray-box attack setting in which the model used to generate the adversarial images is the same as the image-classification model, viz. the ResNet-101; and 2) a black-box attack setting in which the adversarial images are generated using the ResNet-152 model; The backend prediction model of gray-box and black-box is ResNet-101 with different implementations of the state of the art defense methods, such as IGR Ross and Doshi-Velez (2017), PAT Madry et al. (2017), RAT Araujo et al. (2019),Randomization Xie et al. (2017), ALP Kannan et al. (2018), FD Xie et al. (2018) and ADP Pang et al. (2019).

### 5.4 Results and Discussion

**FIGURE 3:**
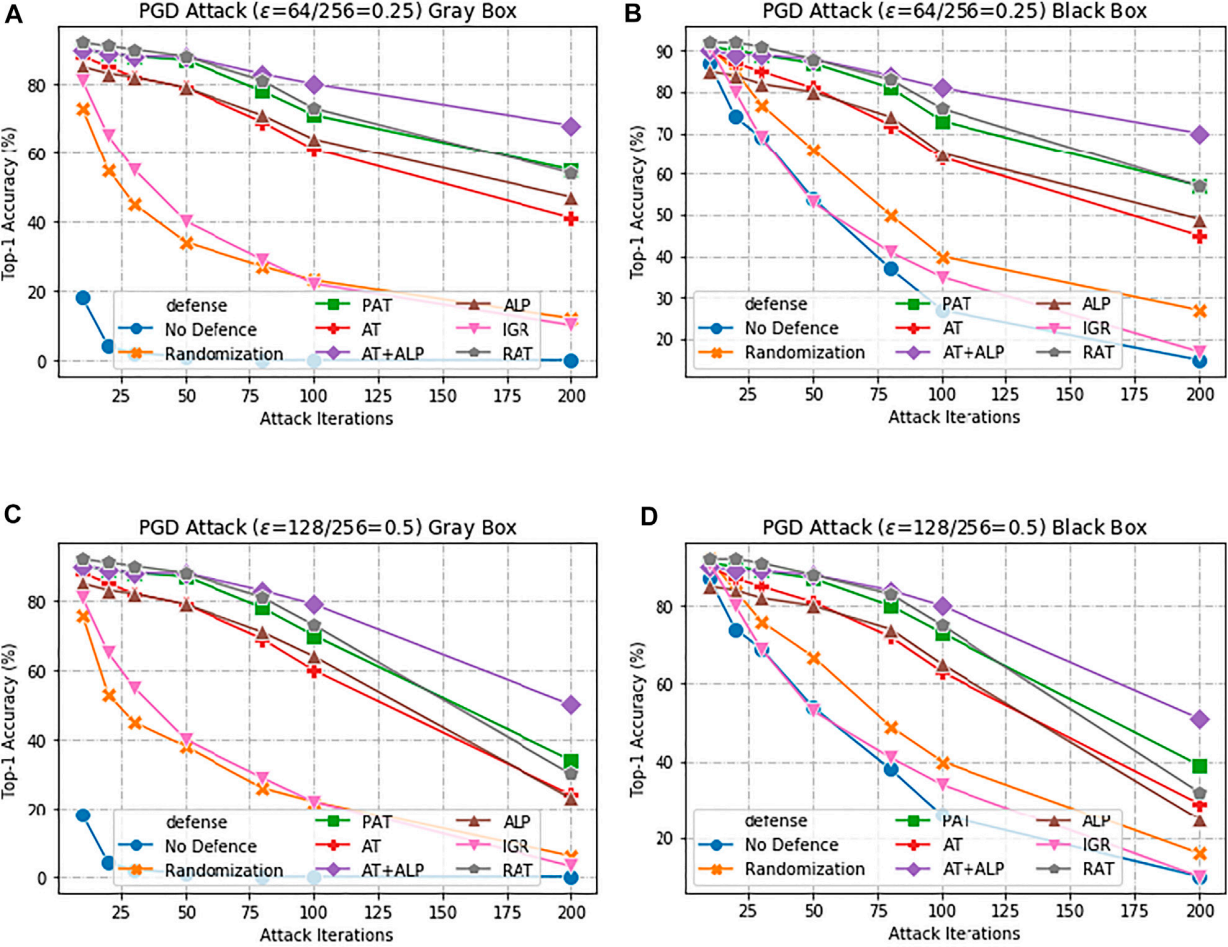
Defense against gray-box and black-box attacks on 17 Flower Category Database. **(A,C)** shows results against a gray-box PGD attacker with 10–200 attack iterations. **(B,D)** shows results against a black-box PGD attacker with 10–200 attack iterations. The maximum perturbation is *ϵ* ∈ {0.25, 0.5}, i.e., *L*_*∞*_ ∈ {0.25, 0.5}.Our **AT + ALP** (purple line) outperform the state-of-the-art in adversarial robustness against highly challenging gray-box and black-box PGD attacks.

Here, we first present results with **AT + ALP** on **17 Flower Category Database**. Compared with previous work, Kannan et al. (2018) was evaluated under 10-iteration PGD attack and *ϵ* = 0.0625, our work are evaluated under highly challenging PGD attack:the maximum perturbation *ϵ* ∈ {0.25, 0.5}, i.e., *L*_*∞*_ ∈ {0.25, 0.5} with 10–200 attack iterations. The bigger the value of *ϵ*, the bigger the disturbance, the more significant the adversarial image effect is. To the best of our knowledge, such a strong attack has not been previously explored on a wide range of datasets. As shown in Figure 3 that **our AT + ALP outperform the state-of-the-art in adversarial robustness against highly challenging gray-box and black-box PGD attacks**. For example, under strong 200-iteration **PGD** gray-box and black-box attacks where prior art has 34 and 39% accuracy, our method achieves **50** and **51%**.

Table 2 shows **Main Result** of our work: under strong 200-iteration PGD gray-box and black-box attacks, **our AT + ALP outperform the state-of-the-art in adversarial robustness on all these databases**.

**TABLE 2:** Defense against gray-box and black-box attacks on 17 Flower Category Database, Part of ImageNet Database and Dogs-vs.-Cats Database. The adversarial perturbation were produced using PGD with step size *α* = 1.0/256 = 0.0039 and 200 attack iterations. As shown in this table, **AT + ALP got the highest Top-1 Accuracy on all these database**.

17 flower category database	Gray-box		Black-box	
*ε* = *L*_*∞*_	0.25	0.5	0.25	0.5
No Defence	0	0	15	10
IGR Ross and Doshi-Velez (2017)	10	3	17	10
PAT Madry et al. (2017)	55	34	57	39
RAT Araujo et al. (2019)	54	30	57	32
Randomization Xie et al. (2017)	12	6	27	16
ALP Kannan et al. (2018)	47	23	49	25
FD Xie et al. (2018)	33	10	33	10
ADP Pang et al. (2019)	22	8	23	8
Our **AT**	41	24	45	29
Our **AT + ALP**	68	50	70	51

**Part of ImageNet database**	**Gray-box**		**Black-box**	
***ε*** **=** ***L***_***∞***_	**0.25**	**0.5**	**0.25**	**0.5**
No Defence	2	3	52	50
IGR Ross and Doshi-Velez (2017)	32	32	34	34
PAT Madry et al. (2017)	76	76	77	77
RAT Araujo et al. (2019)	76	76	77	76
Randomization Xie et al. (2017)	40	41	62	59
ALP Kannan et al. (2018)	54	54	55	55
FD Xie et al. (2018)	60	61	61	61
ADP Pang et al. (2019)	42	44	43	44
Our **AT**	76	76	77	76
Our **AT + ALP**	82	82	82	82

**Dogs-vs.-Cats Database**	**Gray-box**		**Black-box**	
***ε*** **=** ***L***_***∞***_	**0.25**	**0.5**	**0.25**	**0.5**
No Defence	1	1	52	53
IGR Ross and Doshi-Velez (2017)	57	60	51	52
PAT Madry et al. (2017)	51	51	52	52
RAT Araujo et al. (2019)	49	49	50	50
Randomization Xie et al. (2017)	10	8	55	54
ALP Kannan et al. (2018)	57	56	57	57
FD Xie et al. (2018)	57	57	57	57
ADP Pang et al. (2019)	50	50	50	50
Our **AT**	50	50	50	50
Our **AT + ALP**	67	67	71	71

**FIGURE 4:**
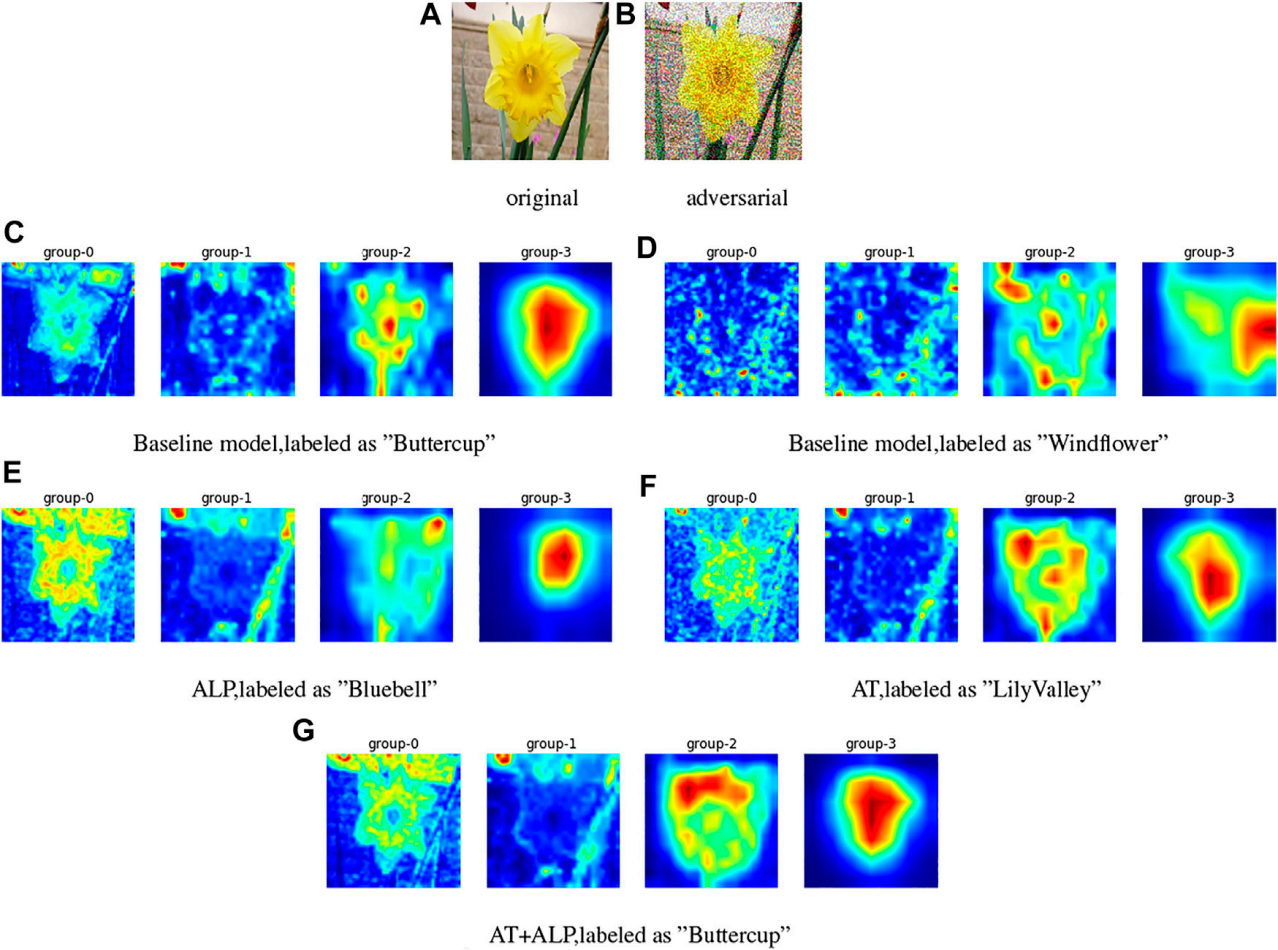
Activation attention maps for defense against gray-box PGD attacks (*ϵ* = 0.25) on 17 Flower Category Database. **(A)** is original image and **(B)** is corresponding adversarial image. **(C)** and **(D)** are activation attention maps of baseline model for original image and adversarial image, **(E,F,G)** are activation attention maps of **ALP**, **AT** and **AT + ALP** for adversarial image. Group-0 to group-3 represent the activation attention maps of four groups of convolutional structures in the baseline model, group-0 extracts of low-level features, group-1 and group-2 extract of mid-level features, group-3 extracts of high-level features Zagoruyko and Komodakis (2016). It can be clearly found that group-0 of AT + ALP can extract the outline and texture of flowers more accurately, and group-3 has a higher level of activation on the whole flower, compared with other defense methods, only it makes accurate prediction.

We visualized activation attention maps for defense against PGD attacks. Baseline model is ResNet-101 He et al. (2015), which is pre-trained on **ImageNet** Russakovsky et al. (2015) and fine-tuned on **17 Flower Category Database** Nilsback and Zisserman (2006), group-0 to group-3 represent the activation attention maps of four groups of convolutional structures in the baseline model, i.e., *conv*2_*x*, *conv*3_*x*, *conv*4_*x* and *conv*5_*x* of ResNet-101, group-0 extracts of low-level features, group-1 and group-2 extract of mid-level features, group-3 extracts of high-level features Zagoruyko and Komodakis (2016);. We found from Figure 4 that group-0 of **AT + ALP** can extract the outline and texture of flowers more accurately, and group-3 has a higher level of activation on the whole flower, compared with other defense methods, only **AT + ALP** makes accurate prediction.

**FIGURE 5:**
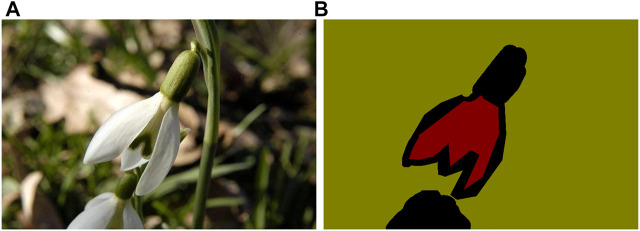
**(A)** is original image and **(B)** is corresponding discriminative parts. **17 Flower Category Database** defined discriminative parts of flowers. So for each image, we got several key regions which are very important to discriminate its category.

**FIGURE 6:**
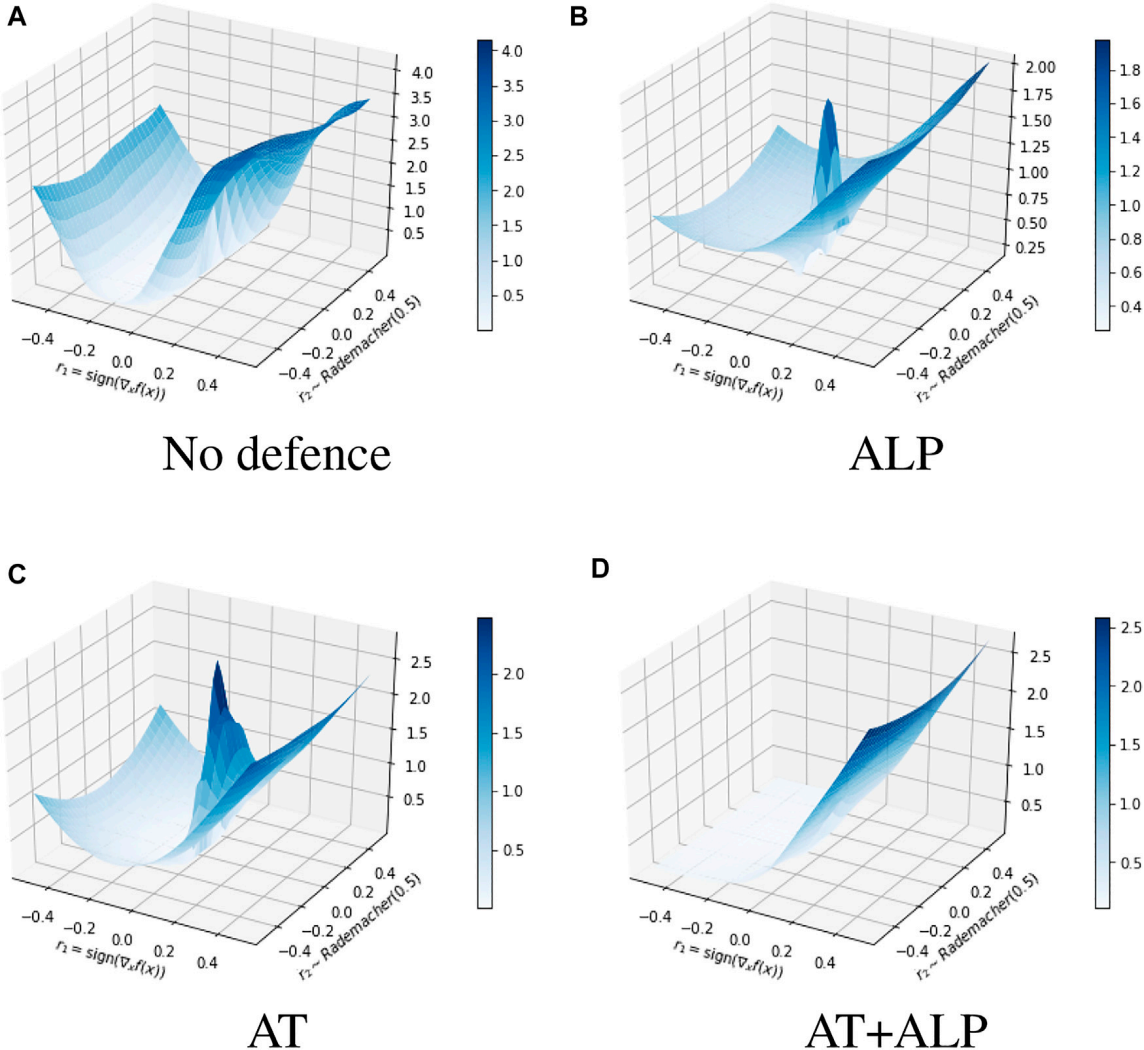
Comparison of loss landscapes generated by No defence **(A)**, ALP **(B)**, AT **(C)** and AT+ALP **(D)**. We see that **ALP** and **AT** sometimes induces decreased loss near the input locally, and gives a “bumpier” optimization landscape, our **AT + ALP** has better robustness. The *z* axis represents the loss. If *x* is the original input, then we plot the loss varying along the space determined by two vectors: $r1=sign\left({▽}_{x}f\left(x\right)\right)$ and *r*2 ∼ *Rademacher* (0.5). We thus plot the following function: *z* = *loss* (*x* ⋅ *r*1 + *y* ⋅ *r*2).

We compared average activations on discriminate parts of **17 Flower Category Database** for different defense methods. **17 Flower Category Database** defined discriminative parts of flowers. See Figure 5 for an illustrative example. These discriminative parts are annotated by humans, according to their contributions to recognize a target. In other words, they are crucial features for the classification. For example, the head and feather should be discriminative parts to recognize a species of bird. Using all testing examples of **17 Flower Category Database**, we calculated normalized activations on these key regions of these different defense methods. As shown in Table 3, **AT + ALP** got the highest average activations on those key regions, demonstrating that **AT + ALP** focused on more discriminate features for flowers recognition. We also demonstrate in Figure 6 that **AT + ALP** shows smoother loss landscapes, which further verifies its effectiveness.

**TABLE 3:** Comparing **average activations** on discriminate parts of **17 Flower Category Database** for different defense methods. In addition, we included new statistical results of activations on part locations of **17 Flower Category Database** supporting the above qualitative cases. The **17 Flower Category Database** defined discriminative parts of flowers. So for each image, we got several key regions which are very important to discriminate its category. Using all testing examples of **17 Flower Category Database**, we calculated normalized activations on these key regions of these different defense methods. As shown in this table, **AT + ALP** got the highest average activations on those key regions, demonstrating that **AT + ALP** focused on more discriminate features for flowers recognition.

Defense	Black-box		Gray-box	
*ε* = *L*_*∞*_	0.25	0.5	0.25	0.5
No Defense	0.41	0.41	0.21	0.21
ALP Kannan et al. (2018)	0.16	0.16	0.15	0.15
IGR Ross and Doshi-Velez (2017)	0.37	0.37	0.33	0.33
PAT Madry et al. (2017)	0.42	0.42	0.44	0.44
RAT Araujo et al. (2019)	0.40	0.40	0.41	0.41
Our **AT**	0.55	0.54	0.56	0.56
Our **AT + ALP**	0.98	0.98	0.96	0.96

## 6 Conclusion

In this paper, we introduced enhanced defense using a technique we called **Attention and Adversarial Logit Pairing (AT + ALP)**, a method that encouraged both attention map and logit for pairs of examples to be similar. When being applied to clean examples and their adversarial counterparts, **AT + ALP** improved accuracy on adversarial examples over adversarial training. Our **AT + ALP** achieves **the state of the art** defense on a wide range of datasets against **PGD** gray-box and black-box attacks. Compared with other defense methods, our **AT + ALP** is simple and effective, without modifying the model structure, and without adding additional image preprocessing steps.

## Data Availability

The original contributions presented in the study are included in the article/Supplementary Material, further inquiries can be directed to the corresponding authors.

## Footnotes

1
https://www.kaggle.com/chetankv/dogs-cats-images
